# Enhancing UAV Object Detection in Low-Light Conditions with ELS-YOLO: A Lightweight Model Based on Improved YOLOv11

**DOI:** 10.3390/s25144463

**Published:** 2025-07-17

**Authors:** Tianhang Weng, Xiaopeng Niu

**Affiliations:** School of Computer Science and Artificial Intelligence, Beijing Technology and Business University, Beijing 100048, China; 2330702051@st.btbu.edu.cn

**Keywords:** low-light conditions, YOLOv11, lightweight, model pruning

## Abstract

Drone-view object detection models operating under low-light conditions face several challenges, such as object scale variations, high image noise, and limited computational resources. Existing models often struggle to balance accuracy and lightweight architecture. This paper introduces ELS-YOLO, a lightweight object detection model tailored for low-light environments, built upon the YOLOv11s framework. ELS-YOLO features a re-parameterized backbone (ER-HGNetV2) with integrated Re-parameterized Convolution and Efficient Channel Attention mechanisms, a Lightweight Feature Selection Pyramid Network (LFSPN) for multi-scale object detection, and a Shared Convolution Separate Batch Normalization Head (SCSHead) to reduce computational complexity. Layer-Adaptive Magnitude-Based Pruning (LAMP) is employed to compress the model size. Experiments on the ExDark and DroneVehicle datasets demonstrate that ELS-YOLO achieves high detection accuracy with a compact model. Here, we show that ELS-YOLO attains a mAP@0.5 of 74.3% and 68.7% on the ExDark and DroneVehicle datasets, respectively, while maintaining real-time inference capability.

## 1. Introduction

Drone-view object detection (DVOD) aims to locate and classify objects in images or videos captured by unmanned aerial vehicles (UAVs) [1]. With the rapid advancements in computer vision and UAV technologies, DVOD has become prevalent in diverse applications, including security surveillance, intelligent transportation systems, and environmental monitoring, achieving significant results. For instance, Wu et al. [2] proposed CCR-Net, a multimodal feature fusion network that improves operational efficiency in disaster response and emergency relief missions. Huang et al. [3] developed UFPMP-Det, which accurately identifies crop diseases and pests from UAV imagery. Zhan et al. [4] introduced ARGNet, effectively detecting forest fire smoke. Hu et al. [5] proposed CM-YOLO, which enhances the detection performance of aircraft and ships under cloud and mist conditions. Peng et al. [6] designed the MMDLTH framework to achieve robust detection of small infrared targets against heavy cloud clutter. By integrating deep learning algorithms, UAV systems can monitor critical events such as traffic violations and accidents in real time, providing essential support for informed decision making.

Nighttime security patrols and search-and-rescue missions require real-time and precise monitoring over large regions. However, traditional manual inspections are inefficient and prone to missed detections. Conventional surveillance equipment struggles with capturing detailed imagery under low-light conditions and lacks automated data analysis capabilities. UAV systems equipped with object detection algorithms offer rapid deployment, mobility, and automated recognition, enabling efficient wide-area monitoring in a short time [7]. Therefore, developing robust and efficient object detection techniques for low-light conditions has become a critical research focus [8].

Currently, most object detection methods for low-light conditions primarily rely on image preprocessing techniques, such as brightness adjustment and noise suppression, to enhance image quality and thereby improve detection performance [9]. For example, Guo et al. [10] proposed an illumination map estimation method that initializes low-light images based on maximum RGB channel values. Hu et al. [11] mitigated color distortion in low-light images through saturation adjustment. Jeon et al. [12] combined atmospheric scattering models with pixel-adaptive gamma correction for image enhancement. However, these enhancement-based methods exhibit inherent limitations. First, the enhancement process can introduce artifacts that obscure essential image details. Second, reliance on fixed prior knowledge restricts adaptability to dynamically changing lighting conditions and limits the model’s ability to learn deeper, high-level semantic features. Moreover, the computational overhead associated with image enhancement is significant for resource-constrained edge devices, severely limiting their real-time performance and practical deployment.

The YOLO (You Only Look Once) [13] series models represent single-stage object detection frameworks capable of performing localization and classification simultaneously in a single forward pass. These models, characterized by simple architectures, effectively balance detection accuracy and real-time performance, making them suitable for resource-constrained edge devices. Lightweight variants such as YOLOv8-nano, YOLOv9-tiny [14], and YOLOv10-nano [15] demonstrate robust performance on natural image datasets like Pascal VOC [16] and MS COCO [17], but they are not optimized specifically for low-light or UAV-captured imagery. Consequently, their performance significantly deteriorates under complex backgrounds and weak object features.

To address these issues, we propose a lightweight object detection model tailored specifically for low-light conditions called ELS-YOLO. This model builds upon the YOLOv11s framework and aims to balance detection accuracy with architectural efficiency.

The main contributions of this work are summarized as follows:

1. We design the re-parameterized backbone ER-HGNetV2, which integrates Re-parameterized Convolution (RepConv) [18] and Efficient Channel Attention (ECA) [19] mechanisms to effectively capture high-quality features, suppress noise, and enhance feature representation in low-light environments.
2. We develop LFSPN, which enables efficient cross-scale feature fusion and improves both model generalization and detection capability across diverse object scales.
3. We propose SCSHead, a lightweight detection head that leverages shared convolutions with separate batch normalization layers to minimize computational complexity and enhance inference efficiency. Furthermore, we incorporate Layer-Adaptive Magnitude-Based Pruning (LAMP) [20] to precisely prune redundant parameters, thereby reducing computational costs without compromising detection performance.
4. Extensive experiments conducted on the ExDark and DroneVehicle datasets demonstrate that ELS-YOLO achieves an optimal balance between detection accuracy and inference speed, validating its practical deployment potential.

## 2. Background

### 2.1. DVOD: Drone-View Object Detection

As an emerging research direction in remote sensing, DVOD faces unique challenges compared with conventional ground-view detection. Images captured by drones often contain numerous targets with significant scale variations, complicating detection accuracy and robustness. Existing DVOD approaches can be broadly classified into three categories: super-resolution-based, context-based, and representation fusion-based methods.

Super-resolution-based methods [21,22] enhance small object detectability by reconstructing low-resolution regions into high-resolution representations, typically through a three-stage pipeline: candidate region proposal, super-resolution reconstruction, and detection. Although these methods significantly improve object perception, their multi-stage structures often introduce redundant computation and complicate the training process, limiting their practicality and end-to-end optimization capability.

Context-based methods [23,24] leverage local and global contextual information to build spatial relationships and semantic dependencies, enhancing semantic representation and scene understanding. However, drone imagery often presents complex backgrounds and ambiguous semantic boundaries, hindering effective context modeling and reducing overall detection accuracy.

Representation fusion-based methods [25,26] integrate fine-grained spatial details from shallow features with high-level semantic features from deeper layers, primarily using architectures such as the Feature Pyramid Network (FPN) and its variants. Nonetheless, under low-light conditions, the representational gap between scales is pronounced, and direct fusion may introduce noise, degrading the discriminative ability of the model.

In addition, object detection from the UAV perspective faces multiple challenges arising from environmental factors. First, high-speed flight often results in image blur and motion trails, which significantly increase the difficulty of real-time detection. Second, weather variations such as fog, rain, and wind can degrade image quality and affect flight stability, thereby impairing object perception. Third, UAVs are susceptible to both intentional and unintentional electromagnetic interference, which may cause motor stalling, sensor drift, or even communication link failures [27,28]. All these factors can adversely impact the real-time performance and accuracy of object detection systems.

### 2.2. LLOD: Low-Light Object Detection

Existing research on object detection in low-light environments mainly focuses on improving image quality through low-light image enhancement (LLIE) and enhancing detection performance through architectural optimization.

LLIE techniques aim to restore critical information in dark regions by enhancing image brightness, contrast, and overall visual quality. Early LLIE methods relied on pixel intensity mapping and local statistical modeling. Techniques such as exposure correction [29] adjust global brightness distributions to enhance visibility, whereas histogram equalization [30] redistributes pixel intensity histograms to increase contrast. The Retinex theory [31] offers a physically interpretable enhancement framework by decomposing an image into illumination and reflectance components, thereby modeling contributions from lighting and surface texture. In recent years, deep neural networks have achieved significant advancements in LLIE tasks. LLNet [32] was the first deep autoencoder-based network designed for simultaneous low-light enhancement and denoising. Wei et al. [33] combined the Retinex theory with convolutional neural networks, incorporating Gaussian filtering and logarithmic transformation to perform adaptive brightness correction. Guo et al. proposed Zero-DCE [34,35], a method that achieves fast, reference-free image enhancement by learning pixel-wise luminance adjustment curves. Xu et al. [36] developed an SNR-aware network that adaptively enhances images through global attention mechanisms and local structure modeling.

Architectural optimization aims to improve feature extraction and object recognition under low-light conditions by refining network structures and incorporating attention mechanisms. Long et al. [37] proposed a multi-level illumination learning framework, SCINet, which enhances feature extraction under complex backgrounds. Qiu et al. [38] introduced Efficient Attention Pyramid Transformer (EAPT), which integrates deformable attention and a global encoder–decoder structure to improve multi-scale feature modeling. Hu et al. [39] proposed an occlusion-aware attention module, MPCM, to alleviate detection difficulties caused by occlusion. Peng et al. [40] enhanced detection performance in low-light scenarios by optimizing attention mechanisms and the loss function. Wu et al. [41] developed the progressive enhancement network AENet, combining Yeo–Johnson transformation with a Transformer architecture to improve dynamic feature representation. Wang et al. [42] and Li et al. [43] mitigated the challenges posed by complex environments by employing geometry-aware learning and advanced signal reconstruction techniques.

## 3. Baseline Algorithm

**Figure 1:**
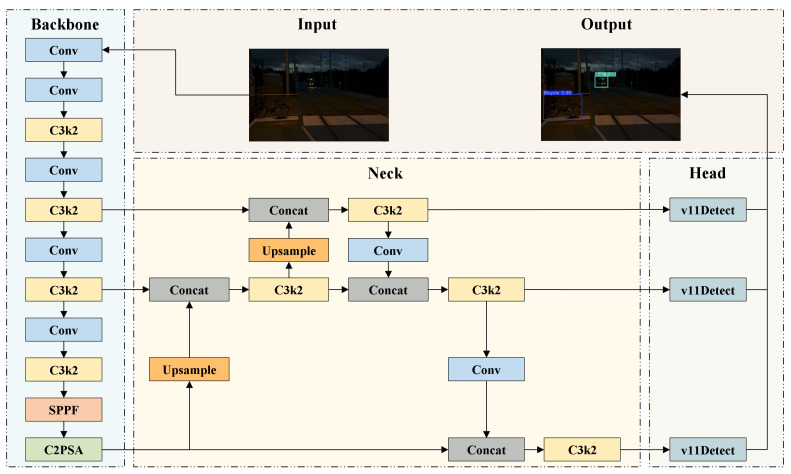
Overall architecture of the YOLOv11 model.

YOLOv11 [44] is the latest version of the YOLO series, and provides five model variants—n, s, m, l, and x—to support deployment across a spectrum of platforms, ranging from edge devices to high-performance servers. As illustrated in Figure 1, YOLOv11 adopts a classic three-stage architecture consisting of a backbone, neck, and head, which are responsible for feature extraction, feature fusion, and object detection, respectively.

**Figure 2:**
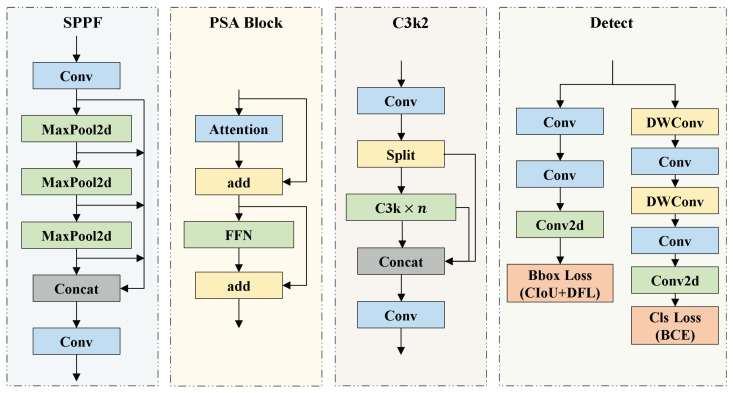
Detailed architectural design of core modules in YOLOv11.

As a significant advancement in real-time object detection, YOLOv11 inherits the high efficiency and end-to-end detection capabilities of previous YOLO models, while incorporating multiple architectural innovations and optimizations. An overview of YOLOv11 primary modules is presented in Figure 2. The C3k2 module serves as the core structural unit of YOLOv11 and adjusts the kernel size by modifying the C3k parameter to meet the feature extraction requirements of different scenarios. The C2PSA module uses spatial attention to guide the model to focus on key regions and improve the accuracy of small or occluded objects. In the detection head, YOLOv11 employs depthwise separable convolutions (DWConv) to replace standard convolutions, thereby further reducing the parameter count and accelerating inference speed.

We selected YOLOv11s as the baseline due to its superior performance under identical experimental conditions. As demonstrated in Table 1 and Table 2, YOLOv11s achieved an optimal balance between detection accuracy, parameter count, and computational complexity on the ExDark dataset relative to other YOLO variants. Moreover, compared with the “n” variants, the “s” versions provided higher detection precision with only a slight increase in computational overhead, making them particularly suitable for lightweight UAV deployments.

## 4. Methodology

### 4.1. ER-HGNetV2: Re-Parameterized Backbone

**Figure 3:**
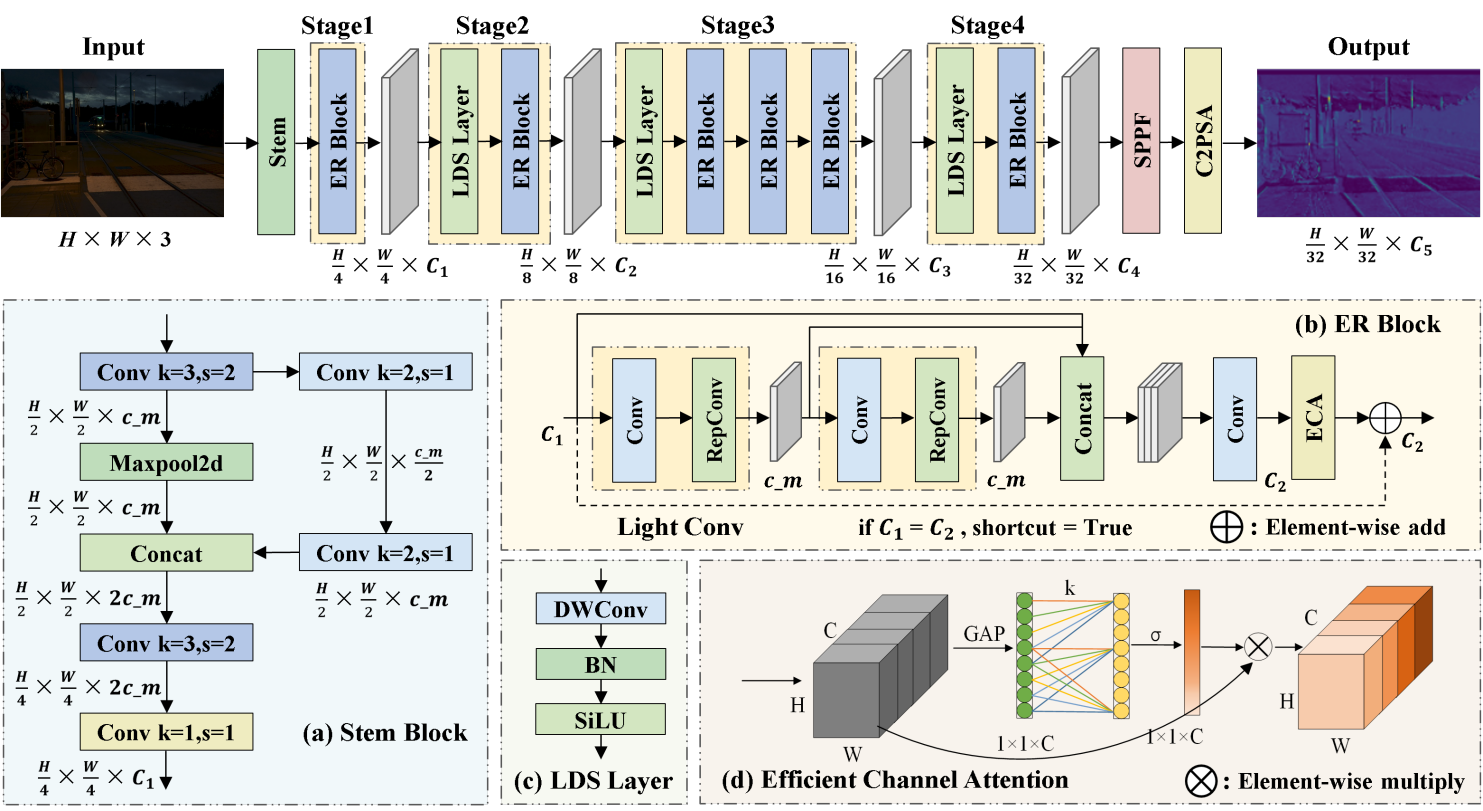
Network architecture of the proposed ER-HGNetV2 backbone.

The backbone network of YOLOv11 primarily consists of alternating stacks of standard convolutional layers and C3k2 modules. Although this design demonstrates strong feature extraction capabilities, its increasing depth and channel width lead to parameter redundancy and substantial computational overhead. Moreover, this structure struggles to effectively capture complex global semantic features in low-light UAV imagery. To address this issue, inspired by the HGNetV2 framework [45], we design a re-parameterized backbone, ER-HGNetV2, which is illustrated in Figure 3.

ER-HGNetV2 begins with a Stem module that consists of standard convolution and max-pooling operations, performing initial spatial downsampling and extracting fundamental feature representations. ERBlock forms the core of the network and applies a multi-scale feature extraction strategy based on stacked convolution layers to refine features and enhance representational accuracy. The LDS layer integrates Depthwise Convolution (DWConv), batch normalization, and SiLU activation, enabling independent channel-wise convolution that enhances feature expressiveness while maintaining minimal computational overhead.

**Figure 4:**
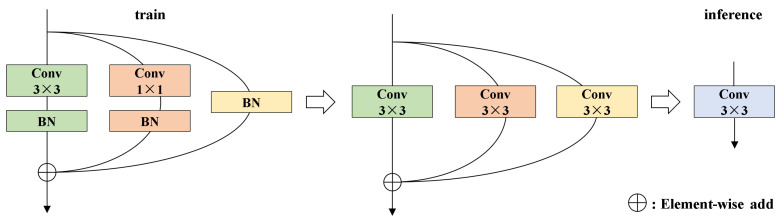
Re-parameterization process of the RepConv module.

We construct ERBlock using the RepConv and ECA mechanism. As illustrated in Figure 4, RepConv adopts a multi-branch training structure that incorporates 3×3 convolutions, 1×1 convolutions, and identity mappings to capture diverse feature patterns. During inference, RepConv applies re-parameterization to fuse convolutional layers and batch normalization into a single standard convolution, thereby achieving a balance between representational capacity and inference efficiency. As shown in Figure 3, the ECA mechanism leverages a lightweight 1D convolution to adaptively compute channel-wise attention weights. This compact design effectively emphasizes critical feature channels with minimal computational cost, thereby improving the model’s ability to distinguish targets under low-light conditions.

### 4.2. LFSPN: Lightweight Feature Selection Pyramid Network

**Figure 5:**
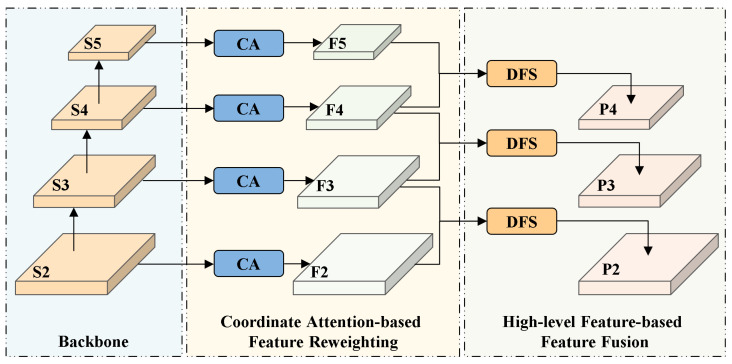
Architecture of the proposed Lightweight Feature Selection Pyramid Network.

The Path Aggregation Feature Pyramid Network (PAFPN) used in YOLOv11 suffers from unselective aggregation and cross-level semantic inconsistency during feature fusion, making it difficult to obtain discriminative multi-scale representations. To address this issue, we design the LFSPN, which selectively strengthens semantically relevant features and suppresses redundant background information to significantly improve model robustness under challenging lighting conditions. As shown in Figure 5, LFSPN consists of two stages: an attention-weighted stage and a dynamic cross-scale feature fusion stage.

**Figure 6:**
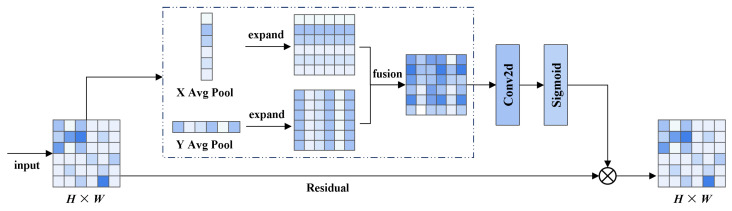
Illustration of the CA mechanism.

The attention-weighted stage performs initial feature selection on the multi-scale feature maps extracted by the backbone and enhances spatial position awareness via the Coordinate Attention (CA) [46] mechanism. As shown in Figure 6, the CA mechanism first applies global average pooling along the horizontal and vertical directions to capture spatial dependencies. It then concatenates the pooled features along the channel dimension and applies a 1×1 convolution to model cross-directional spatial relationships and generate intermediate attention weights. Finally, pixel-wise weighting is applied to the original feature map using the generated attention weights to emphasize informative regions and suppress redundant features, improving the quality of representations for the subsequent fusion stage.

**Figure 7:**
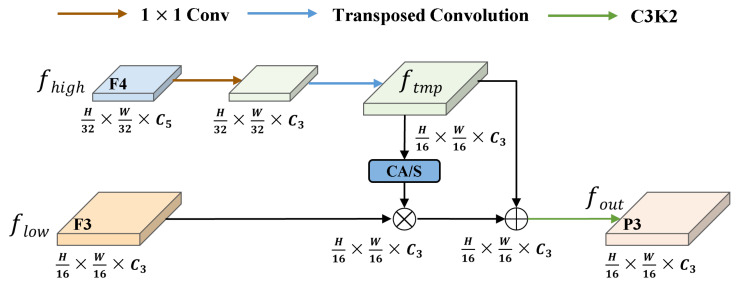
Framework of the Dynamic Feature Selection module.

To further improve the effectiveness of feature fusion, we design a Dynamic Feature Selection (DFS) module, as shown in Figure 7. Taking the high-level feature map $F_{4}$ and the low-level feature map $F_{3}$ as an example, we first apply a 1×1 convolution to $F_{4}$ for nonlinear dimensionality reduction to lower computational cost. Then we upsample $F_{4}$ using transposed convolution to match the spatial resolution of $F_{3}$. The CA mechanism is applied to the upsampled feature map $F_{4}$ to generate attention weights for dynamic feature selection. These weights are element-wise multiplied with $F_{3}$ to selectively enhance spatially informative regions in the low-level feature map. The refined $F_{3}$ is then fused with $F_{4}$ through element-wise addition to produce a feature representation that preserves both fine-grained spatial details and high-level semantic information. The fused features are passed through a C3k2 module to further enhance feature representations, resulting in the final output feature map, as

(1) $$f_{\mathrm{tmp}}=\mathrm{Trans}\left({\mathrm{Conv}}_{1\times 1}\left(f_{\mathrm{high}}\right)\right)$$

(2) $$f_{\mathrm{out}}=C3k2\left(f_{\mathrm{low}}\cdot \mathrm{CA}\left(f_{\mathrm{tmp}}\right)+f_{\mathrm{tmp}}\right)$$

### 4.3. SCSHead: Shared Convolution and Separate Batch Normalization Head

**Figure 8:**
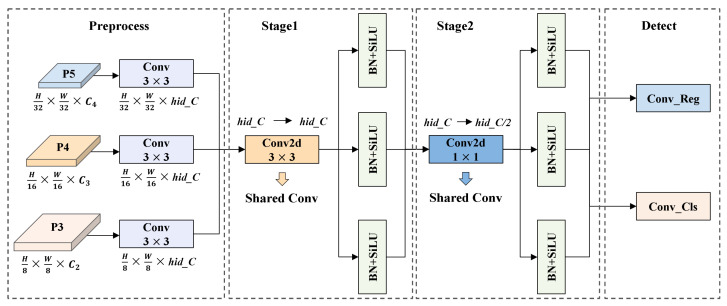
Architecture of the proposed SCSHead.

On UAV and other edge devices, detection head must balance high accuracy and low computational cost. However, conventional designs typically use separate convolutional branches for each prediction task, which leads to parameter redundancy. To address this issue, we propose the SCSHead, as shown in Figure 8.

SCSHead adopts a shared-weight convolutional architecture. Feature maps from different scales are first processed by individual 3×3 convolutional layers to align their channel dimensions, thereby ensuring consistent cross-scale feature representation. The aligned feature maps are then passed through a shared-weight convolutional module consisting of two successive convolutional stages. First, a 3×3 convolutional layer is applied to extract cross-scale features and enhance representational capacity. Subsequently, a 1×1 convolutional layer performs channel-wise compression and reorganization, thereby reducing the number of parameters and computational cost. Finally, the processed features are fed into two parallel convolutional layers, Conv_Reg for predicting bounding box coordinates and Conv_Cls for classification scores.

To accommodate statistical discrepancies across feature maps of different scales, we introduce a scale-specific normalization strategy. Specifically, each input branch applies an independent BN layer. This design allows each scale to adaptively normalize its feature statistics, thereby preserving and emphasizing the discriminative properties of individual feature maps.

### 4.4. Network Structure of ELS-YOLO

**Figure 9:**
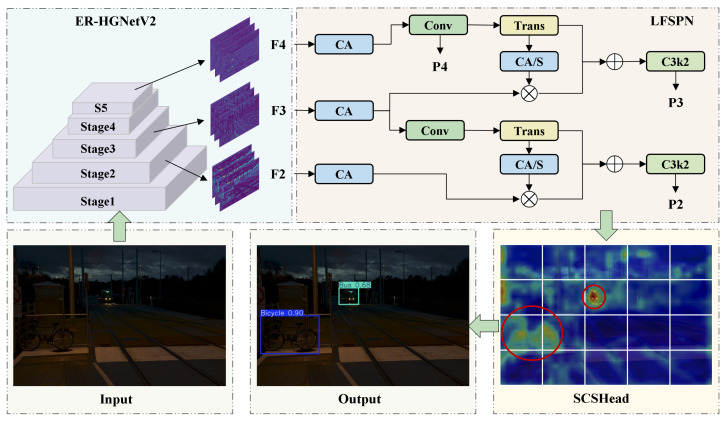
Overall architecture of the proposed ELS-YOLO model.

The overall architecture of the proposed ELS-YOLO is shown in Figure 9. The input image is first processed by the ER-HGNetV2 backbone to extract hierarchical features. These features are passed through LFSPN for dynamic cross-scale fusion to improve multi-scale representation. The fused features are then sent to SCSHead to produce the final detection results.

### 4.5. Channel Pruning

Deploying object detection systems on UAV platforms necessitates real-time processing of aerial imagery to accurately identify and localize traffic signs, vehicles, pedestrians, and other critical targets. However, UAVs are typically constrained by limited computational resources, memory, and battery capacity. Therefore, detection models must be carefully optimized to reduce both computational complexity and memory consumption. Model pruning has emerged as an effective structural optimization technique that significantly compresses model size while preserving near-original performance. To further improve the real-time inference capability of the proposed model on edge devices, we apply the LAMP method to eliminate redundant parameters and reduce model complexity.

**Figure 10:**
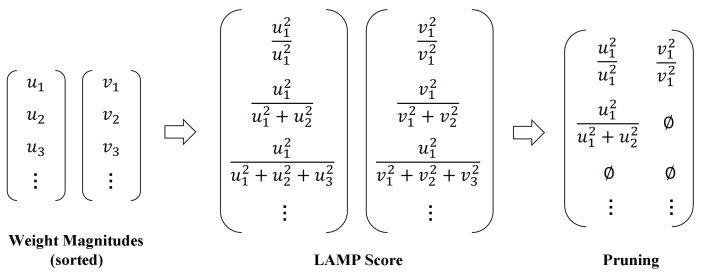
The LAMP pruning process.

Traditional magnitude-based pruning methods apply a global threshold to prune all layers of the network uniformly. However, such approaches fail to account for the varying importance of parameters across layers, often resulting in excessive pruning of critical layers and subsequent degradation in model accuracy. To address this issue, the LAMP method introduces a layer-wise adaptive importance scoring mechanism. By reweighting the importance of weights within each layer, LAMP determines an appropriate pruning ratio for each layer. As shown in Figure 10, for a given layer’s weight tensor *W*, its values are first flattened into a one-dimensional vector and sorted in ascending order based on magnitude. Based on this sorted vector, LAMP assigns each weight *u* a corresponding score that reflects its relative importance within the layer, as

(3) $$\mathrm{score}\left(u,W\right)=\frac{{\left(W\left[u\right]\right)}^{2}}{\sum_{v\geq u}{\left(W\left[v\right]\right)}^{2}}$$

where the numerator represents the squared value of the current weight, and the denominator denotes the sum of squares of all weights whose magnitudes are greater than or equal to that of the current weight.

The LAMP score reflects the relative contribution of each weight among the remaining connections. During pruning, the model removes connections with lower scores on a per-layer basis until the desired global sparsity level is reached. This strategy accounts for the varying contributions of different layers to the overall network, thereby enabling substantial model compression while preserving detection accuracy to the greatest extent possible.

## 5. Experimental Results

### 5.1. Dataset

To evaluate the effectiveness and generalizability of the proposed ELS-YOLO model for object detection in low-light conditions, we conducted extensive experiments on two publicly available datasets: ExDark [47] and DroneVehicle [26]. We randomly split the dataset into training, validation, and test sets in an 8:1:1 ratio.

#### 5.1.1. ExDark

**Figure 11:**
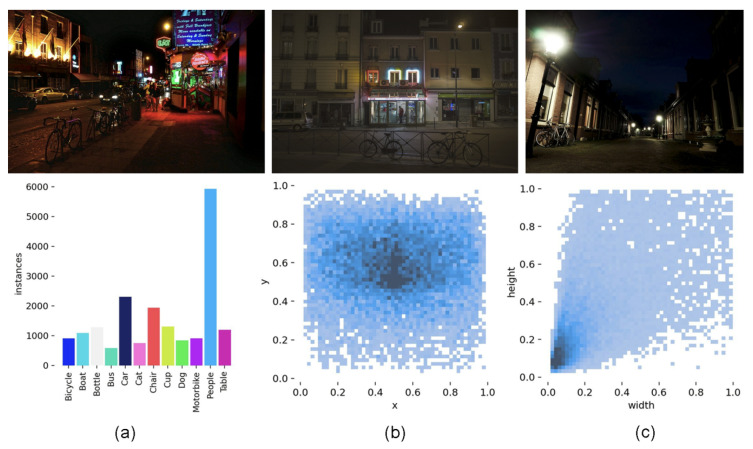
Illustration and statistical analysis of the ExDark dataset. (**a**) Class distribution of annotated objects, (**b**) distribution of normalized bounding box sizes, and (**c**) spatial distribution of bounding box center points across the image plane.

The ExDark dataset is designed for object detection under low-light conditions. It contains 7363 real-world images captured in various challenging lighting environments and provides annotations for 12 object categories. Figure 11 shows typical image examples and a statistical overview of the annotations.

#### 5.1.2. DroneVehicle

**Figure 12:**
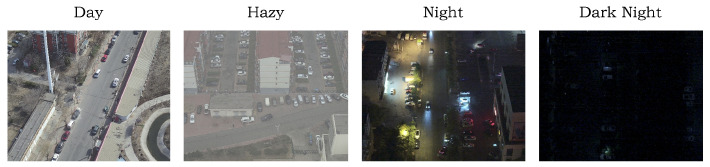
Images under varying illumination conditions.

The DroneVehicle dataset contains aerial images captured by UAVs across various urban scenes such as city roads, residential areas, parking lots, and highways. The images are categorized into four lighting conditions: Day, Hazy, Night, and Dark Night. Figure 12 shows typical examples under each condition. Since this study focuses on object detection in low-light environments, we retain only the images labeled as Night and Dark Night.

### 5.2. Experimental Environment

The experiment is conducted using Python 3.10.16 and PyTorch 2.3.1 on an NVIDIA GeForce RTX 4090 GPU. We use SGD as the optimizer with an initial learning rate of 0.01, momentum set to 0.937, and weight decay of 0.0005. Each model is trained for 200 epochs with a batch size of 16 and no pretrained weights. All comparison experiments use the same settings.

### 5.3. Evaluation Indicators

The performance of the proposed model is evaluated using commonly adopted object detection metrics, including precision, recall, mean average precision (mAP), frames per second (FPS), and GFLOPs. The definitions of these metrics are presented as follows:

(4) $$\mathrm{Precision}=\frac{TP}{TP+FP}$$

(5) $$\mathrm{Recall}=\frac{TP}{TP+FN}$$

(6) $$AP=\int_{0}^{1}\mathrm{Precision}\left(\mathrm{Recall}\right)\;d\left(\mathrm{Recall}\right)$$

(7) $$\mathrm{mAP}=\frac{1}{K}\sum_{i=1}^{K}AP_{i}$$

where TP denotes the number of correctly detected positive samples, FP denotes the number of falsely detected positive samples, FN denotes the number of ground truth objects that were missed by the detector, *K* denotes the total number of object categories in the dataset, and $AP_{i}$ represents the average precision for the *i*-th category.

### 5.4. Experimental Analysis on the ExDark Dataset

#### 5.4.1. ER-HGNetV2 Experiment

To validate the effectiveness of the proposed ER-HGNetV2 backbone for low-light object detection, we conducted comparative experiments using YOLOv11s as the baseline and by replacing its backbone with ER-HGNetV2 and other existing lightweight alternatives. Table 3 presents the experimental results on the ExDark dataset.

The proposed ER-HGNetV2 backbone demonstrates clear advantages across all evaluation metrics, achieving the highest scores in both mAP@0.5 and mAP. Compared with the original HGNetV2, ER-HGNetV2 yields better detection performance while incurring lower computational complexity.

#### 5.4.2. Comparison with YOLOv11

To evaluate the performance advantages of ELS-YOLO over the YOLOv11 series models, we conducted a quantitative analysis on the ExDark dataset. The experimental results are presented in Table 1.

**Figure 13:**
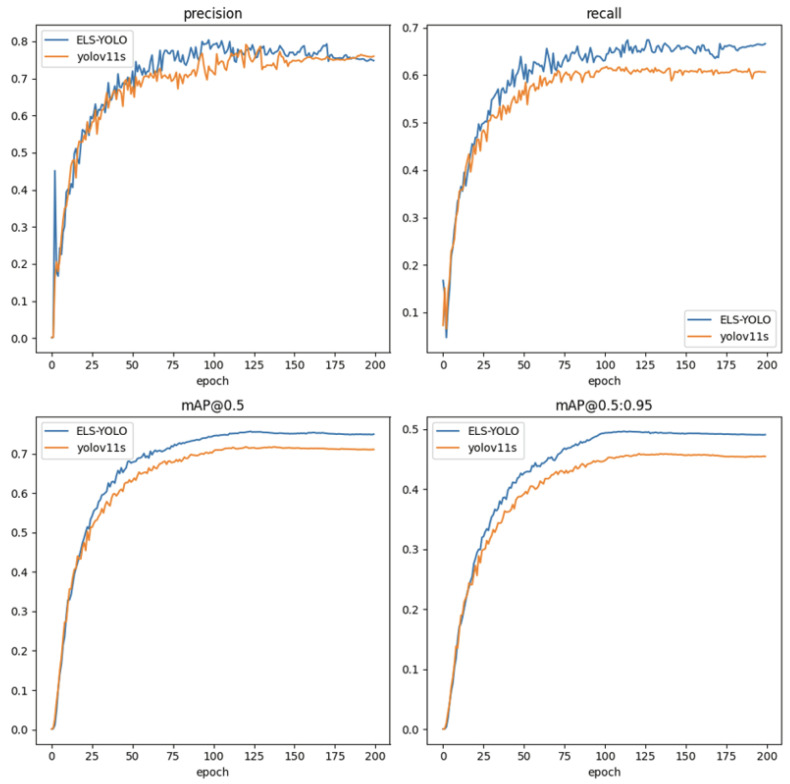
Training curves of ELS-YOLO and YOLOv11s.

In terms of detection performance, ELS-YOLO achieves a mAP@0.5 of 74.3% and mAP of 48.5%. Compared with the baseline model YOLOv11s, ELS-YOLO improves mAP@0.5 by 2.9% and mAP by 2.8%, demonstrating stronger robustness in low-light conditions. Regarding model complexity, ELS-YOLO contains only 48.9% of the parameters and 70.4% of the computational cost of YOLOv11s, demonstrating excellent lightweight characteristics. Figure 13 illustrates the training performance curves of four key metrics for both ELS-YOLO and YOLOv11s. The proposed ELS-YOLO outperforms YOLOv11s across all four metrics throughout the training process.

#### 5.4.3. LAMP Experiment

We conducted a detailed analysis of the ELS-YOLO model’s performance under different pruning ratios. The pruning ratio is defined as the ratio of computational cost before pruning to that after pruning. For example, a pruning ratio of 1.33 indicates a 25% reduction in computation, a ratio of 2 indicates a 50% reduction, and a ratio of 4 indicates a 75% reduction.

As shown in Table 4, when the pruning ratio is set to 1.33, the number of parameters is reduced from 4.6 M to 2.4 M, corresponding to a compression rate of 47.8%. In terms of detection performance, mAP@0.5 remains at 74.3%, consistent with the unpruned model, while mAP decreases by only 0.1%. When the pruning ratio is increased to 2.0, the model achieves an optimal balance between compression and performance. Specifically, the parameter count further decreases to 1.3M, and mAP@0.5 is maintained at 74.2%. However, when the pruning ratio increases to 4.0, although the number of parameters continues to decline, detection performance drops sharply, with mAP@0.5 decreasing to 62.4% and mAP falling to 37.5%.

Based on the above experimental results, the pruning ratio is set to 2.0, which provides the best trade-off between model compactness and detection accuracy.

#### 5.4.4. Ablation Experiments

To comprehensively assess the contribution of each key component in the proposed ELS-YOLO, we conducted a series of ablation experiments. The corresponding experimental results are summarized in Table 5.

First, we replace the original backbone with the proposed ER-HGNetV2 to enhance the model’s ability to represent and generalize target features under low-light conditions. As shown in Table 5, this substitution resulted in a 1.2 increase in the mAP@0.5 metric, while the computational complexity was reduced by 3 GFLOPs. These results indicate that ER-HGNetV2 effectively eliminates redundant computation and improves the network’s capacity to extract complex features in dark environments.

Next, we apply the proposed SCSHead, which further increases mAP@0.5 to 73.8%. This result indicates that SCSHead, through its shared convolutional structure and scale-adaptive normalization, enhances the network’s robustness and discriminative capacity for multi-scale object detection in low-light conditions.

Finally, we introduce LFSPN to enhance multi-scale feature fusion. The addition of LFSPN improved mAP@0.5 and mAP by 0.5% and 0.6%, respectively. Meanwhile, the model’s parameter and computational complexity were significantly reduced. These results confirm that LFSPN effectively integrates multi-scale information while suppressing redundant features, thereby improving both the network’s generalization capability and computational efficiency.

#### 5.4.5. Comparison Experiments with Other Baseline Methods

To further evaluate the effectiveness and performance advantages of the proposed ELS-YOLO, we conduct comparison experiments under the same settings with several mainstream detection models.

As presented in Table 2, ELS-YOLO demonstrates clear advantages across all key performance metrics, outperforming lightweight YOLO variants and DETR-based models. In terms of model complexity, ELS-YOLO maintains a compact architecture with only 4.6 M parameters and 15.0 GFLOPs, which is significantly lower than that of larger models such as Faster R-CNN and DETR. These results further support the feasibility of deploying ELS-YOLO for real-time inference on resource-constrained edge devices.

#### 5.4.6. Visualization Analysis

To intuitively evaluate the detection performance of the proposed ELS-YOLO under low-light conditions, four representative scenes from the ExDark dataset were selected. These scenes encompass various challenges, including small object detection, multi-scale targets, severe occlusion, and complex lighting environments.

**Figure 14:**
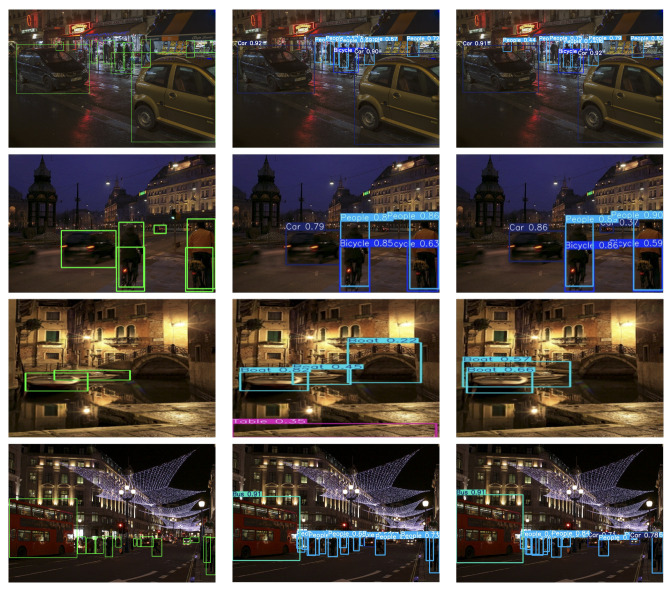
Detection results of different models on the ExDark dataset. From left to right: ground truth, YOLOv11s, and ELS-YOLO.

From the visualization results in Figure 14, it is evident that ELS-YOLO demonstrates superior object detection and localization capabilities compared with YOLOv11s. Specifically, in the first row, under extremely poor illumination, ELS-YOLO accurately detects partially occluded targets in dim lighting conditions. In the second row, which depicts a distant and weakly illuminated scene, ELS-YOLO effectively detects small-scale pedestrians and vehicles, whereas YOLOv11s exhibits clear omissions. These results suggest that the ER-HGNetV2 backbone and the LFSPN structure enhance ELS-YOLO’s ability to capture fine-grained features and small object information in low-light environments. In the third row, representing a multi-scale detection scenario, YOLOv11s shows noticeable false detections. In contrast, ELS-YOLO successfully identifies multiple object instances with higher precision and better localization, highlighting the benefits of the proposed SCSHead in cross-scale feature sharing and adaptive normalization. Additionally, in the fourth row, an urban night scene, ELS-YOLO provides clear and accurate detections across objects of various scales. In comparison, YOLOv11s performs poorly on distant targets, leading to both false negatives and false positives.

**Figure 15:**
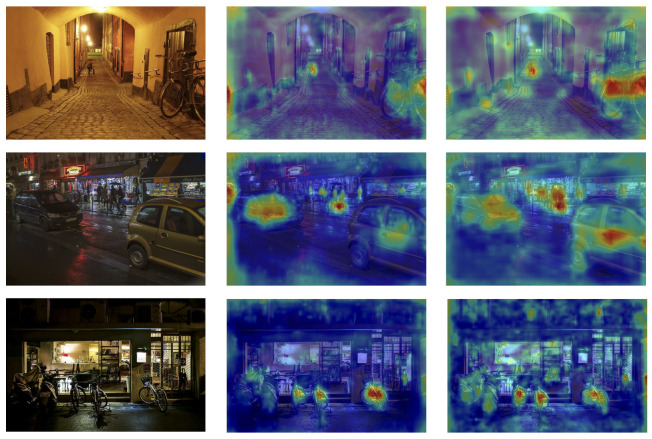
Heatmap results. From left to right: original images, YOLOv11s, and ELS-YOLO.

We use Gradient-weighted Class Activation Mapping (Grad-CAM) [52] to visualize the focus regions of both ELS-YOLO and the baseline YOLOv11s during prediction. As shown in Figure 15, ELS-YOLO exhibits more accurate attention to critical object regions compared with YOLOv11s. In the first row, under a dimly lit scene, YOLOv11s displays weak activation for distant pedestrians and bicycles. In contrast, ELS-YOLO significantly enhances attention to these small objects. In the second row, which depicts a complex nighttime environment, YOLOv11s either overlooks or diffusely attends to vehicles and distant pedestrians. However, ELS-YOLO produces more concentrated attention centered on the key regions of the targets. The third row further confirms the robustness and superiority of ELS-YOLO. Under partial occlusion, YOLOv11s generates vague and dispersed attention, whereas ELS-YOLO accurately highlights the partially occluded small objects.

### 5.5. Experimental Analysis on the DroneVehicle Dataset

**Figure 16:**
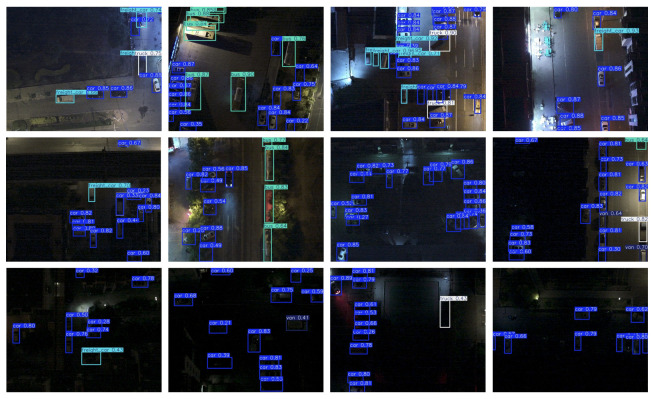
Detection results of ELS-YOLO on the DroneVehicle dataset.

To further assess the performance of the proposed ELS-YOLO model in DVOD tasks, we conducted experiments on the DroneVehicle dataset. This dataset comprises aerial images captured by UAVs in various urban scenarios, including city roads, residential areas, parking lots, and highways. Representative inference results are presented in Figure 16, and the corresponding performance comparison is summarized in Table 6.

As shown in Table 6, ELS-YOLO achieves a precision of 68.3%, recall of 67.5%, mAP@0.5 of 68.7%, and mAP of 44.5%. Compared with the baseline model YOLOv11s, ELS-YOLO improves mAP@0.5 by 1.5% and mAP by 1.6%, demonstrating superior target localization capabilities.

## 6. Discussion

We conducted extensive experiments on the representative ExDark and DroneVehicle datasets to evaluate the object detection performance of ELS-YOLO under complex low-light environments. These datasets include diverse low-light scenarios and drone perspectives characterized by substantial scale variations and complex backgrounds. The experimental results indicate that ELS-YOLO achieves a mAP@0.5 of 74.3% and 68.7% on ExDark and DroneVehicle, respectively, significantly surpassing YOLOv11s and other mainstream detection models. This performance enhancement is primarily attributed to structural innovations, resulting in superior capabilities in feature extraction and fusion strategies. Compared with other lightweight models, ELS-YOLO exhibits advantages in parameter scale, computational complexity, and inference efficiency, highlighting its potential for deployment in edge computing environments.

Although ELS-YOLO demonstrates strong overall performance, there remains room for improvement under certain conditions. For instance, its detection accuracy still needs enhancement in scenarios involving extreme low-light conditions or severe occlusion. In addition, although this study has significantly reduced the model’s parameter size, further improving inference speed without sacrificing accuracy remains an important direction for future research.

## 7. Conclusions

In this paper, we propose the lightweight object detection model ELS-YOLO to address the challenges of limited detection performance in complex low-light conditions and constrained computational resources on edge devices. We design the re-parameterized backbone ER-HGNetV2 to enhance the ability to extract and represent critical features under low-light environments. To overcome the limitations of conventional fusion strategies, we introduce LFSPN, which enables efficient multi-scale feature integration. We also develop the lightweight detection head SCSHead to reduce computational cost and parameter count. Furthermore, we apply the LAMP pruning strategy to compress model size without sacrificing accuracy. Extensive experiments on the ExDark and DroneVehicle datasets demonstrate that ELS-YOLO achieves superior detection accuracy and real-time inference efficiency compared with existing lightweight models.

In future work, we will further explore optimization strategies for the model under extreme conditions, such as incorporating advanced attention mechanisms or dynamic feature fusion methods, to enhance the model’s adaptability in complex environments. Additionally, we will investigate integrating techniques like knowledge distillation and quantization training to further improve the real-time inference capability of the model, thereby facilitating the deployment and application of object detection technologies in a broader range of practical scenarios.

## Figures and Tables

**Table 1:** Comparison with the YOLOv11 series on the ExDark dataset.

Models	mAP@0.5/%	mAP/%	Params/M	GFLOPs/G	FPS
YOLO11n	67.6	42.2	**2.6**	**6.3**	**282**
YOLO11s	71.4	45.7	9.4	21.3	251
YOLO11m	73.2	47.7	20.0	67.7	218
YOLO11l	74.6	48.9	25.2	86.6	183
YOLO11x	**75.7**	**49.7**	56.8	194.5	169
ELS-YOLO	74.3	48.5	4.6	15.0	274

The best value for each metric is shown in bold, and the second-best value is underlined.

**Table 2:** Comparison with other models on the ExDark dataset.

Models	P/%	R/%	mAP@0.5/%	mAP/%	Params/M	GFLOPs/G
YOLOv8n	70.2	59.6	65.7	41.1	3.0	8.1
YOLOv8s	73.9	62.7	70.4	44.3	11.1	28.5
YOLOv9t	74.0	56.7	65.2	40.8	**2.0**	**7.6**
YOLOv9s	74.1	62.1	69.8	44.8	7.2	26.8
YOLOv10n	71.8	58.1	65.0	40.5	2.7	8.2
YOLOv10s	77.2	60.2	69.0	43.8	8.1	24.5
Faster R-CNN	67.4	52.6	58.9	35.2	41.2	208
RetinaNet	66.3	50.7	57.6	33.9	36.5	210
DETR	71.9	57.3	63.8	39.7	40.8	86.2
RT-DETR-r50	75.4	61.5	67.1	42.2	41.9	125.7
RT-DETR-L	73.1	58.1	64.6	39.9	32.0	103.5
ELS-YOLO	**79.2**	**65.8**	**74.3**	**48.5**	4.6	15.0

The best value for each metric is shown in bold, and the second-best value is underlined.

**Table 3:** Comparative experiments with different backbone networks.

Backbone	mAP@0.5/%	mAP/%	Params/M	GFLOPs/G	FPS
baseline	71.4	45.7	9.4	21.3	251
EfficientViT [48]	68.5	43.1	7.98	19.0	214
RepViT [49]	69.3	43.9	10.14	23.5	201
HGNetV2	69.7	44.6	7.61	18.9	220
MobileNetV4 [50]	66.3	41.9	9.53	27.8	**267**
StarNet [51]	65.8	40.1	8.63	**17.6**	174
ER-HGNetV2	**72.6**	**46.5**	**7.6**	18.3	255

The best value for each metric is shown in bold, and the second-best value is underlined.

**Table 4:** Model performance under different pruning rates.

Models	mAP@0.5/%	mAP/%	Params/M	GFLOPs/G	FPS
ELS-YOLO	**74.3**	**48.5**	4.6	15.0	274
ELS-YOLO (ratio = 1.33)	**74.3**	48.4	2.4	11.2	283
ELS-YOLO (ratio = 2.0)	74.2	48.1	1.3	7.4	298
ELS-YOLO (ratio = 4.0)	62.4	37.5	**0.5**	**3.7**	**359**

The best value for each metric is shown in bold, and the second-best value is underlined.

**Table 5:** Ablation experiments performed with the proposed ELS-YOLO.

Models	mAP@0.5/%	mAP/%	Params/M	GFLOPs/G	FPS
baseline	71.4	45.7	9.4	21.3	251
+A	72.6	46.5	7.6	18.3	255
+B	72.2	46.2	9.03	20.4	253
+C	72.7	45.9	6.64	18.7	262
+A+B	73.8	47.9	7.3	17.6	268
+A+C	73.5	47.6	4.84	15.5	271
+A+B+C	**74.3**	**48.5**	**4.6**	**15.0**	**274**

A denotes ER-HGNetV2, B denotes SCSHead, and C denotes LFSPN. The best value for each metric is shown in bold, and the second-best value is underlined.

**Table 6:** Comparative experiments of different object detection algorithms on the DroneVehicle dataset.

Models	P/%	R/%	mAP@0.5/%	mAP/%
YOLO11n	58.4	58.9	61.7	38.6
YOLO11s	**68.2**	63.7	67.2	42.9
RT-DETR-r50	65.7	63.2	66.7	41.2
RT-DETR-L	67.9	66.4	68.1	43.3
ELS-YOLO	68.3	**67.5**	**68.7**	**44.5**
ELS-YOLO (ratio = 2.0)	68.2	67.3	68.5	44.2

The best value for each metric is shown in bold, and the second-best value is underlined.

## Data Availability

The data presented in this study are available from the corresponding author upon reasonable request.
